# Modeling the potential economic benefits of an oral SARS-CoV-2 vaccine during an outbreak of COVID-19

**DOI:** 10.1186/s12889-022-14148-y

**Published:** 2022-09-22

**Authors:** Bryan Patenaude, Jeromie Ballreich

**Affiliations:** 1 Department of International Health, Johns Hopkins Bloomberg School of Public Health, 615 North Wolfe Street, Baltimore, MD 21205 USA; 2 Department of Health Policy and Management, Johns Hopkins Bloomberg School of Public Health, 624 North Broadway, Baltimore, MD 21205 USA

**Keywords:** COVID-19, Vaccines, Economic evaluation, Health economics

## Abstract

**Background:**

Given patient preferences, the choice of delivery modality for vaccines against SARS-CoV-2 has the potential to significantly impact both health and economic consequences of an outbreak of COVID-19. This study models the projected health and economic impact of an oral COVID-19 vaccine in the United States during an outbreak occurring between December 1, 2021 and February 16, 2022.

**Methods:**

A cost-of-illness economic decision analysis model is utilized to assess both the health and economic impact of an oral vaccine delivery platform compared with the status quo deployment of existing intramuscular vaccines against COVID-19. Health impact is assessed in terms of predicted cases, deaths, hospitalization days, intensive care unit admission days, and mechanical ventilation days averted. Health system economic impact is assessed based on the cost-of-illness averted derived from the average daily costs of medical care, stratified by severity. Productivity loss due to premature death is estimated based on regulatory analysis guidelines proposed by the U.S. Department of Health and Human Services.

**Results:**

Based upon preference data, we estimate that the availability of an oral COVID-19 vaccine would increase vaccine uptake from 214 million people to 232 million people. This higher vaccination rate was estimated to result in 2,497,087 fewer infections, 25,709 fewer deaths, 1,365,497 fewer hospitalization days, 186,714 fewer Intensive Care Unit (ICU) days, and 80,814 fewer patient days requiring mechanical ventilation (MV) compared with the status quo. From a health systems perspective, this translates into $3.3 billion in health sector costs averted. An additional $139-$450 billion could have been averted in productivity loss due to a reduction in premature deaths.

**Conclusions:**

Vaccine delivery modalities that are aligned with patient preferences have the ability to increase vaccination uptake and reduce both the health and economic impact of an outbreak of COVID-19. We estimate that the total economic impact of productivity loss and health systems cost-of-illness averted from an oral vaccine could range from 0.6%-2.9% of 2021 U.S, Gross Domestic Product (GDP).

## Background

The Omicron (B.1.1.529) variant of COVID-19 was first reported to the World Health Organization (WHO) on November 24, 2021 from a sample taken in South Africa [1]. Since that time the variant has been found in at least 165 countries and all 50 states within the United States (U.S.), becoming the dominant strain of COVID-19 in the U.S. in December of 2021 and by January of 2022 accounting for more than 99% of incident cases in the US [2, 3]. The rapid spread of Omicron variant is due to its 18,261 unique mutations, over 30 of which occur on the receptor-binding domain of the spike protein, which make it significantly more infectious than the previous B.1.617.2 (Delta) variant and also more likely to evade immunity brought on through both vaccination and previous infection [4]. A recent study shows that these mutations contribute to Omicron variant being approximately three times more infectious than Delta variant and nearly 13-fold more infectious than previous named variants [5, 6]. As of January 2022, Omicron variant was named the second most infectious virus in history with reports of a basic reproductive number, a measure of the average infectiousness of a disease, between 7 and 14 – or one infected individual would be expected, on average to infect 7–14 other individuals [4, 7]. These ranges are only slightly smaller than those observed for the Measles virus, the most infectious diseases ever documented with a basic reproductive number commonly between 12 and 18 [8].

As a result of the high degree of infectiousness of the Omicron variant, COVID-19 cases across the globe began to spike in December and January of 2022, with a peak in reported cases to the US Centers for Disease Control and Prevention (CDC) reaching a seven-day moving average peak of 806,170 new cases per day on January 15, 2022, over three times higher than the highest reported seven-day moving average of 250,341 new cases per day reached on January 11, 2021 and nearly 5 times higher than the 164,477 seven-day moving average of new cases per day peak reached on September 1, 2021 [9]. Throughout this period, hospitalizations averaged nearly 93,000 people per day, with nearly 14,000 people in intensive care units on any given day, and approximately 5,500 people on mechanical ventilators [9]. Given the estimates for the average cost of a hospitalization, intensive care unit (ICU) stay, and an ICU stay with mechanical ventilation (MV) at $11,000, $13,000, and $41,000 respectively, the Omicron variant represents a significant economic burden to the U.S. healthcare system [10].

While several studies have shown that existing vaccines have lower effectiveness against transmission and the development of any infection due to Omicron variant, there is also evidence that existing vaccines, and in particular booster doses of mRNA vaccines, have a significant impact on reducing disease severity with an estimated 90% lower probability of being hospitalized following a booster dose compared with not having received any vaccine [1, 4, 5, 9]. As a result, improvements to the vaccination rate in the U.S. are likely to have substantial impacts on disease severity and the healthcare spending associated with treatment in the context of an outbreak.

One method for increasing the uptake of vaccination in the U.S. is to offer delivery modalities that conform to patient preferences more so than injectable multi-dose vaccines [11]. Several studies have shown that in the U.S., oral vaccines are considered more desirable by patients and a recent study estimated that an oral vaccine against COVID-19 may increase vaccination rates among the unvaccinated by 32%, a population accounting for 92% of the 5,938,358 hospitalization days, 93% of the 817,829 ICU days, and 93% of the 355,276 mechanical ventilation days observed over the December 2021—February 2022 outbreak [11–14]. One such oral vaccine delivery platform is the Vector-Adjuvant-Antigen Standardized Technology (VAAST) platform, developed by Vaxart, Inc. [15]. This study utilizes data on the VAAST platform, paired with both epidemiologic and cost-of-illness data on the most recent Omicron outbreak in the U.S., to estimate the epidemiological and economic impact of an oral vaccine delivery modality as a complement to existing vaccination strategies.

## Methods

To estimate the impact of an oral vaccine on the epidemiologic and economic burden of Omicron variant during an outbreak in the U.S., we utilized a cost-of-illness framework and developed an economic decision tree analysis model. The model compares the epidemiologic and economic burden under the status quo scenario in the United States to two intervention scenarios where an oral vaccine is available. The status quo scenario is defined based on outcomes realized under the existing mix of intramuscular COVID-19 vaccines available between December 2021 and February 2022 utilizing data on cases, deaths, and hospitalizations recorded by the US CDC during the study time period. The intervention scenario is a daily decision tree model utilizing incremental increases in vaccinated based upon oral vaccine revealed preference data. The difference between the status quo and intervention scenarios produces an estimate of the impact of an oral vaccine on the health and economic burden of the Omicron variant compared with existing vaccine delivery modalities. Two scenarios were included for the oral vaccine intervention scenario. The first assumes that that the oral vaccine is equally effective as the existing mix of intramuscular vaccines and performs similarly against transmission, therefore isolating only uptake changes as translating to health impact. The second scenario, based upon clinical evidence, presents a scenario where the oral vaccine is an additional 50% effective against transmission and any infection compared with the existing mix of intramuscular vaccines in addition to the increased uptake rates observed in scenario 2 when compared with intramuscular vaccines [16, 17].

### Epidemiological inputs

Our model was parameterized utilizing data on cases, deaths, and severity of infection, stratified by vaccination status along with cumulative rates of full immunization and booster doses delivered between December 1, 2021 and February 16, 2022, derived from the CDC [9]. The model utilizes stated preference data for an oral vaccine among the unvaccinated population, paired with data on the relative risk of case severity between vaccinated, unvaccinated, and boosted populations to estimate the cases, deaths, and hospitalizations averted through the hypothetical introduction of an oral vaccine during the same timeframe [18]. Data on the relative risks of infection, death, and hospitalization were derived from CDC weekly roundup reports [9]. We averaged the relative risks of infection, death, and hospitalization over the model timeframe to calculate a point estimate parameter for these relative risks. Using the data on relative risks, daily vaccination counts, total cases, deaths, and hospitalizations, we then calculated cases, deaths, and hospitalizations by vaccination status for each day of the outbreak period between December 1, 2021 and February 16, 2022. For ICU days and patient days with mechanical ventilation, we assumed the relative risk by vaccination status was identical to that of overall hospitalizations. The daily relative risk parameters define how many individuals in each category, unvaccinated, fully vaccinated (oral vaccine), fully vaccinated (intramuscular vaccine), boosted (intramuscular vaccine), move or remain in the no disease, mild illness, hospitalized, ICU, and mechanical ventilation states each day. The result is a standard decision tree model with arms for each vaccination status defined by vaccinations at baseline and daily vaccination rates, with transition to each disease state over the time period in daily increments. Results are summed over the days to generate the final results. All analyses are conducted in Microsoft Excel for Mac version 16.63.1.

For the status quo scenario, infections are modeled as a function of cases observed in the raw data, adjusted for the cumulative share of the population vaccinated, unvaccinated, or boosted each day. These modeled cases are then allocated across vaccinated, unvaccinated, and boosted populations based on severity as a function of both the cumulative level of cases in the current period as well as the relative risk of death and hospitalization by vaccination status. This approach allows us to utilize the existing trends in daily transmission, adjusted for vaccination status. For the intervention scenario, we utilize preference data to estimate that 18.7 million additional unvaccinated Americans would accept an oral vaccine [18]. We then adjusted the cumulative of the population vaccinated, unvaccinated, or boosted each day under an oral vaccine introduction scenario. Using the same methodology for modeling cases as the status quo scenario, we then allocated cases across vaccinated, unvaccinated, and boosted populations based on severity as a function of both the cumulative level of cases in the current period as well as the relative risk of death and hospitalization by vaccination status under a base assumption that the oral vaccine would offer the same level of efficacy as current intramuscular vaccines. Additionally, in the primary intervention scenario, we assume that an oral vaccine would offer the same protection against transmissibility of COVID-19 as the intramuscular vaccines. To take into account early clinical evidence, we also model a second intervention scenario where marginal oral vaccine cases exhibit a 50% reduction the relative risk of any infection compared with intramuscular vaccines [16, 17].

For modeling the epidemiological burden specific to the Omicron variant relative to other COVID-19 variants, we utilized data on the relative prevalence of different variants in circulation from CDC surveillance data [3, 9]. CDC surveillance data on variants is reported weekly, so we applied a linear trend between weekly data points to estimate the share for any given day between each weekly measure during the modeled time period. We allocated the share of total cases, deaths, hospitalizations, ICUs, and mechanical ventilation stays based on the relative prevalence of the Omicron variant compared to other variants between December 1, 2021 February 16, 2022. Key epidemiological inputs for the model appear in Table 1.

**Table 1:** Key epidemiological inputs

Epidemiological Parameter	
Relative Risk Unvaccinated vs. Fully Vaccinated (Any COVID)	2.4
Relative Risk Unvaccinated vs. Fully Vaccinated (Death)	14
Relative Risk Unvaccinated vs. Fully Vaccinated (Hospitalization)	16
Relative Risk Unvaccinated vs. Fully Vaccinated (ICU)	16
Relative Risk Unvaccinated vs. Fully Vaccinated (MV)	16
Relative Risk Unvaccinated vs. Boosted (Any COVID)	3.2
Relative Risk Unvaccinated vs. Boosted (Death)	41
Relative Risk Unvaccinated vs. Boosted (Hospitalization)	44
Relative Risk Unvaccinated vs. Boosted (ICU)	44
Relative Risk Unvaccinated vs. Boosted (MV)	44

### Cost inputs

We identified the average daily cost estimates for COVID-19 related hospitalization, ICU stay, or stay on mechanical ventilation in the United States from the published literature [10]. Costs were multiplied by total modeled cases for each health outcome to estimate total costs under the status-quo and oral vaccine intervention scenarios. In addition to health care costs, we also model productivity loss due to premature death. To value productivity loss, we applied the inflation-adjusted value of a statistical life (VSL) range estimates for 2021 produced by the U.S. Department of Health & Human Services as recommended in their update on valuing mortality risks in the context of COVID-19 [19]. The high, low, and central VSL estimates can be found in Table 2 alongside the total deaths under each scenario as well as total economic impact from productivity loss. For each scenario we utilize the high and low estimates to present productivity loss ranges.

**Table 2:** Value of statistical life estimates [19]

2021 VSL (Millions of $)	
Low	$5.4
Medium	$11.5
High	$17.5

### Sensitivity analysis

We conducted two types of sensitivity analyses. First, we conducted a univariate analysis to identify influential parameters for our key model outputs. Second, we conducted a statistical probabilistic sensitivity analysis to estimate 95% confidence intervals around our key model outputs. This analysis was conducted using 5,000 simulations using the @Risk Decision Analysis Add-on for Microsoft Excel®. For both analyses, we set statistical distributions for key epidemiological, cost, and vaccine uptake inputs. For the epidemiological and cost inputs, we assumed a beta distribution. For the vaccine uptake input, we assumed a triangular distribution around estimated uptake.

## Results

We estimate that availability of an oral vaccine with equivalent effectiveness to intramuscular vaccines in the U.S. against COVID-19 between December 1, 2021 and February 16, 2022 would have averted approximately 2,803,787 COVID-19 infections, 30,373 deaths, 1,615,914 hospitalization days, 232,124 ICU days, and 101,189 instances requiring mechanical ventilation (MV) associated with any variant compared with the status quo. Limiting the analysis to only the Omicron variant we estimate an oral vaccine to result in 2,497,087 fewer infections, 25,709 fewer deaths, 1,365,497 fewer hospitalization days, 186,714 fewer Intensive Care Unit (ICU) days, and 80,814 fewer patient days requiring mechanical ventilation (MV) compared with the status quo. Complete epidemiologic impact results are presented in Table 3.

**Table 3:** Epidemiologic burden

		**Cases**	**Deaths**	**Hospitalizations**	**ICU**	**MV**
All Variants	Status Quo	29,468,029	137,829	7,276,077	1,061,443	464,382
	Oral Vaccine Scenario 1	26,664,242	107,455	5,660,163	829,319	363,193
	**Averted Scenario 1**	**2,803,787**	**30,374**	**1,615,914**	**232,124**	**101,189**
	Oral Vaccine Scenario 2	19,986,784	100,729	5,343,350	783,151	343,002
	**Averted Scenario 2**	**9,481,245**	**37,100**	**1,932,727**	**278,292**	**121,380**
Omicron	Status Quo	25,769,048	112,322	5,938,358	817,829	355,276
	Oral Vaccine Scenario 1	23,271,961	86,612	4,572,862	631,115	274,461
	**Averted Scenario 1**	**2,497,088**	**25,709**	**1,365,497**	**186,714**	**80,815**
	Oral Vaccine Scenario 2	17,406,323	81,118	4,313,666	595,438	258,970
	**Averted Scenario 2**	**8,362,726**	**31,203**	**1,624,693**	**222,392**	**96,306**

In scenario 2, an oral vaccine which reduces transmission and probability of any infection by 50% compared with to intramuscular vaccines in the U.S. against COVID-19 between December 1, 2021 and February 16, 2022 would have averted approximately 9,481,245 COVID-19 infections, 37,100 deaths, 1,932,727 hospitalization days, 278,292 ICU days, and 121,380 instances requiring mechanical ventilation associated with any variant compared with the status quo. Limiting the analysis to only the Omicron variant we estimate an oral vaccine under this scenario to result in 8,362,726 fewer infections, 31,203 fewer deaths, 1,624,693 fewer hospitalization days, 222,392 fewer ICU days, and 96,306 fewer patient days requiring mechanical ventilation compared with the status quo.

### Economic burden

From a healthcare perspective, we estimate an oral vaccine of equivalent effectiveness to existing intramuscular vaccines to avert approximately $3.9 billion in treatment costs associated with any variant. This breaks down into $2.9 billion from averted hospitalization days, $0.7 billion in averted ICU days, $0.4 billion in averted patient days on mechanical ventilation. Focusing again on only the averted healthcare costs associated with the Omicron variant, we estimate $3.3 billion in total health sector costs averted, which breaks down into $2.4 billion from averted hospitalization days, $0.5 billion in averted ICU days, $0.3 billion in averted patient days on mechanical ventilation. Complete averted healthcare costs by scenario are presented in Table 4.

**Table 4:** Healthcare costs averted

		**Hospitalizations**	**ICU**	**MV**	**Total**
All Variants	Status Quo	$12,893,208,444	$3,080,307,586	$1,752,408,802	$17,725,924,832
	Oral Vaccine Scenario 1	$10,029,809,055	$2,406,683,835	$1,370,556,923	$13,807,049,813
	**Averted Scenario 1**	**$2,863,399,389**	**$673,623,751**	**$381,851,879**	**$3,918,875,019**
	Oral Vaccine Scenario 2	$9,468,415,443	$2,272,703,270	$1,294,364,405	$13,035,483,118
	**Averted Scenario 2**	**$3,424,793,001**	**$807,604,316**	**$458,044,397**	**$4,690,441,714**
Omicron	Status Quo	$10,522,771,115	$2,373,340,366	$1,340,682,366	$14,236,793,848
	Oral Vaccine Scenario 1	$8,103,110,685	$1,831,495,124	$1,035,716,597	$10,970,322,405
	**Averted Scenario 1**	**$2,419,660,431**	**$541,845,242**	**$304,965,770**	**$3,266,471,443**
	Oral Vaccine Scenario 2	$7,643,815,500	$1,727,960,169	$977,257,337	$10,349,033,006
	**Averted Scenario 2**	**$2,878,955,615**	**$645,380,197**	**$363,425,030**	**$3,887,760,841**

For the higher transmission reduction scenario, we estimate an oral vaccine to avert approximately $4.7 billion in treatment costs associated with any variant. This breaks down into $3.4 billion from averted hospitalization days, $0.8 billion in averted ICU days, $0.5 billion in averted patient days on mechanical ventilation. Focusing again on only the averted healthcare costs associated with the Omicron variant, we estimate $3.9 billion in total health sector costs averted, which breaks down into $2.9 billion from averted hospitalization days, $0.6 billion in averted ICU days, $0.4 billion in averted patient days on mechanical ventilation.

Applying a regulatory estimate for the productivity loss associated with premature death, we find that an additional $164-$530 billion in economic benefits could be attained in averted productivity loss due to premature death from any variant, or $139-$450 billion from deaths averted due to Omicron in the equivalent effectiveness scenario. In the secondary reduced transmission scenario we find that an additional $200-$649 billion in economic benefits could be attained in averted productivity loss due to premature death from any variant, or $168-$546 billion from deaths averted due to Omicron. Given a U.S. Gross Domestic Product (GDP) in 2021 of $22.9 trillion, the total economic impact of both productivity loss and health systems costs averted due to an oral vaccine ranged from 0.6%-2.0% of 2021 U.S. GDP in the equivalent effectiveness scenario and 0.8%-2.9% of 2021 U.S. GDP in the reduced transmission scenario [20]. Complete productivity loss results are presented in Table 5.

**Table 5:** Productivity loss averted

		**Deaths**	**Productivity Loss (Low)**	**Productivity Loss (Low)**	**Productivity Loss (High)**
All Variants	Status Quo	137,829	$744,276,600,000	$1,585,033,500,000	$2,412,007,500,000
	Oral Vaccine Scenario 1	107,455	$580,257,526,181	$1,235,733,620,571	$1,880,464,205,216
	**Averted Scenario 1**	**30,374**	**$164,019,073,819**	**$349,299,879,429**	**$531,543,294,784**
	Oral Vaccine Scenario 2	100,729	$543,934,686,154	$1,158,379,424,216	$1,762,751,297,721
	**Averted Scenario 2**	**37,100**	**$200,341,913,846**	**$426,654,075,784**	**$649,256,202,279**
Omicron	Status Quo	112,322	$606,536,502,230	$1,291,698,106,600	$1,965,627,553,522
	Oral Vaccine Scenario 1	86,612	$467,707,425,230	$996,043,590,768	$1,515,718,507,691
	**Averted Scenario 1**	**25,709**	**$138,829,076,999**	**$295,654,515,832**	**$449,909,045,831**
	Oral Vaccine Scenario 2	81,118	$438,038,940,274	$932,860,706,138	$1,419,570,639,775
	**Averted Scenario 2**	**31,203**	**$168,497,561,956**	**$358,837,400,462**	**$546,056,913,747**

### Sensitivity analysis

**Fig. 1:**
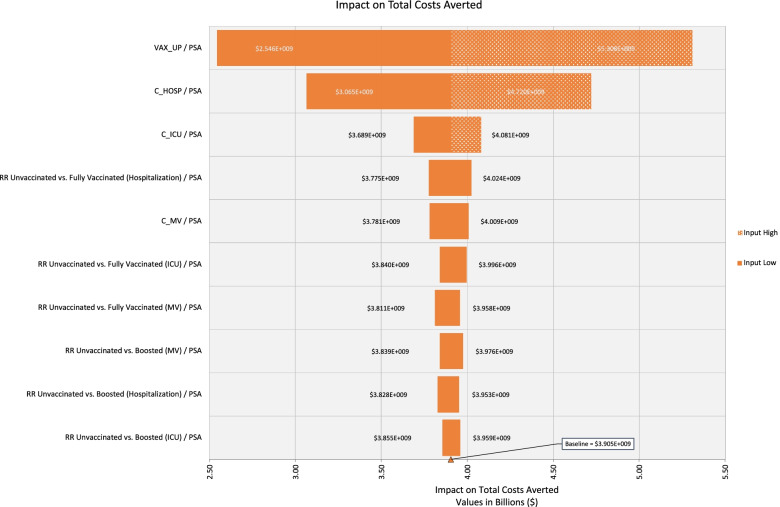
Tornado diagram for total costs averted

For epidemiological outputs such as cases averted, the univariate analysis identified vaccine uptake as the most influential parameter followed by that output’s relative risk parameter. For the total cost averted output, the univariate analysis identified vaccine uptake as the most influential parameter. This was followed by cost of hospitalization and cost of ICU stays. The tornado diagram for total cost averted is presented in Fig. 1.

The probabilistic sensitivity analysis yielded 95% confidence interval estimates around our key outputs to account for model uncertainty due to the varying statistical distributions associated with input parameters. Uncertainty estimates are provided in Table 6.

**Table 6:** Uncertainty estimates around key outputs

		5% Lower Bound	95% Upper Bound
All Variants	Cases Averted Scenario 1	1,690,073	3,925,199
	Cases Averted Scenario 2	8,220,112	10,741,490
Omicron Variant	Cases Averted Scenario 1	1,507,559	3,495,682
	Cases Averted Scenario 2	7,245,243	9,482,724
All Variants	Total Costs Averted Scenario 1	$2,438,601,000	$5,585,059,000
	Total Costs Averted Scenario 2	$3,117,132,000	$6,495,989,000
Omicron Variant	Total Costs Averted Scenario 1	$2,030,328,000	$4,656,985,000
	Total Costs Averted Scenario 2	$2,577,641,000	$5,390,628,000

## Discussion

Our results demonstrate large healthcare cost-savings as well as productivity loss reductions as a result of the availability of an oral vaccine delivery modality in the U.S. in the context of the 2021–2022 Omicron variant outbreak. The primary pathway for the increases in cases and deaths averted from an oral vaccine is through alignment in vaccine delivery modality with patient preferences, thereby increasing vaccination uptake among the currently unvaccinated. The likelihood of this phenomena is backed in the literature as well as market studies, indicating that oral delivery mechanisms are strongly favored over injectables, particularly among the unvaccinated or vaccine hesitant [11–14].

We find that both a scenario where an oral vaccine has equivalent effectiveness to the mix of existing intramuscular vaccines as well as a scenario where oral vaccines disrupt transmission by reducing the likelihood of infection at rate 50% higher than existing oral vaccines have large potential economic benefits. However, the marginal gains in benefits between a reduced transmission scenario and an equivalent effectiveness scenario are smaller than between an equivalent effectiveness scenario and the status quo. This is largely due to a large unvaccinated population and a modest increase in oral vaccination use relative to the large number of previously vaccinated. Had 100% of the population that was already vaccinated as of December 1, 2021 received the reduced transmission scenario’s oral vaccine instead of the existing intramuscular vaccines, we would expect benefits associated with a reduced transmission scenario to be substantially larger.

There are several limitations to our analysis. The first is that we focus our effort on examining the relative impact of vaccination during a specific previous outbreak. While this may reduce the generalizability of outcomes, despite signs of COVID-19 beginning to exit the pandemic and enter into an endemic state, a combination of waning immunity from existing vaccines and rapidly mutating variants that may more readily evade existing immunity, lend support to the likelihood that similar proportional gains in epidemiologic and economic outcomes from an oral vaccine compared with intramuscular vaccines could be expected for future outbreaks [17]. A second limitation is that we exclude the costing of vaccines from the analysis and focus only on quantifying the direct benefits of vaccine delivery modality. While supply and delivery costs based upon know distribution for existing intramuscular vaccines are known, the comparable costs for oral vaccines vary widely and prices for such vaccines as negotiated by health systems are unknown. As an attempt to limit the number of assumptions made, we have chosen to focus our modeling and estimation on the benefits side of a cost–benefit analysis which can be complemented with realistic costing data as that becomes available. Evidence suggests that oral vaccines are likely to result in substantial delivery cost savings over intramuscular vaccines, as well as potential spillover economic benefits from reduction in medical commodities such as syringes as well as medical waste [21]. A third limitation is that we do not have geospatial data on transmissions and as such our model utilizes national averages in terms of transmission and relative risk of infection conditional on vaccination. While we believe this still allows for an accurate comparison of the relative levels of outcomes between status quo and interventions groups, the absolute levels of all groups modeled should be interpreted with caution. Models that address geographic-specific could be considered in future research to provide more nuance to where oral vaccines are likely to have greater relative impact.

## Conclusions

Our estimates suggest that an oral vaccine delivery modality would have a significant impact on both health and economic outcomes by increasing the likelihood of vaccination among the unvaccinated. The benefits observed stem primarily from reductions in the severity of infection and premature mortality among the unvaccinated. Economic impact is driven by fewer hospitalizations and productivity losses averted through prevented deaths. We estimate that the total economic impact of productivity loss and healthcare costs averted from an oral vaccine could range from 0.6%-2.9% of 2021 U.S, GDP. Policymakers should consider oral vaccines as a viable method for reduce the health and economic burden of outbreaks of COVID-19 through aligning vaccine delivery systems with patient preferences.

## Data Availability

The data that support the findings of this study are available from Vaxart, Inc., but restrictions apply to the availability of these data, which were used under license for the current study, and so are not publicly available. Data are however available from the authors upon reasonable request and with permission of Vaxart, Inc.

## Abbreviations

CDC: US Centers for Disease Control and Prevention
COVID-19: Coronavirus Disease 2019
GDP: Gross Domestic Product
ICU: Intensive Care Unit
MV: Mechanical Ventilation
SARS-CoV-2: Severe Acute Respiratory Syndrome Coronavirus 2
U.S.: United States
VAAST: Vector-Adjuvant-Antigen Standardized Technology
VSL: Value of a Statistical Life
WHO: World Health Organization
